# Shared Failures: Uniting Four Career Pathways to Overcome Decentralized Wastewater Workforce Challenges in Limited-Resource Rural Communities

**DOI:** 10.1021/acsestwater.5c00978

**Published:** 2025-11-14

**Authors:** Michel Kordahi, Amal Bakchan

**Affiliations:** Department of Construction Science, 14736 Texas A&M University, 3137 TAMU, College Station, Texas 77843, United States

**Keywords:** Decentralized Wastewater, Infrastructure Management, Workforce, Rural Areas, Limited-Resource Communities, Thematic Analysis

## Abstract

Persistent sanitation gaps in limited-resource rural communities across the United States continue to hinder progress toward national sustainable development goals. Addressing these communities’ wastewater needs has led to a substantial rise in decentralized wastewater systems (DWSs). However, the ongoing nationwide workforce crisis threatens the effectiveness of these systems’ management, including operation and maintenance (O&M). Current literature narrowly frames workforce challenges as labor shortages, overlooking critical and deeper systemic issues. Consequently, policy-informed environmental workforce strategies remain a pressing national need. Here, we develop an integrated cross-pathway approach for achieving sustainable decentralized wastewater workforce development by investigating the root causes of both shortages and shortcomings that emerge across four interconnected career pathways: academic, regulatory, professional, and skilled trade. Through theoretical thematic analysis of 30 semistructured interviews with stakeholders across rural Alabama’s Black Belt, we uncover how misaligned expectations, disconnected responsibilities, and siloed institutions collectively erode the capacity of the DWS workforce. Our findings highlight targeted policy interventions, including state administrative code reforms to promote performance-based regulation, mandate proactive O&M, and strengthen state-led, community-driven engagement. By exposing how fragmented career pathways undermine effective DWS governance in limited-resource rural communities, this work joins ongoing efforts to address complex environmental problems and advance sustainable development.

## Graphical abstract

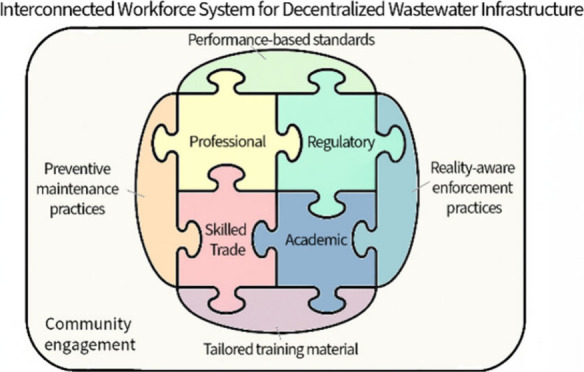

## 1 Introduction

Infrastructure does not fail in isolation—it fails through broken relationships among the people, policies, and professions meant to sustain it. Nowhere is this more evident than in the water and wastewater infrastructure systems in rural communities in the United States (U.S.). Many of these communities continue to struggle with persistent sanitation gaps,^1,2^ hindering progress toward Sustainable Development Goal^3^ (SDG) 6—Clean Water and Sanitation for All. To address these needs, decentralized wastewater systems (DWSs)—including individual onsite treatment systems and clustered systems serving multiple households—have been increasingly deployed and currently serve about 65% of rural U.S. households.^4^ However, rural communities that rely most heavily on DWSs often lack the foundational capacities needed to plan, finance, implement, or maintain these systems. In these communities—referred to hereafter as “*limited-resource rural communities*”—weak governance structures, insufficient local workforce capacity, and limited financial resources exacerbate DWS vulnerabilities.^2^ As a result, ensuring effective and sustainable DWS management is essential for protecting public health, protecting the environment, and advancing national sustainable development efforts.

Beyond technological advancements, effective DWS solutions must account for the financial, social, and institutional conditions that shape infrastructure performance in limited-resource rural communities, ensuring that interventions are sustainable and scalable.^2^ Even when capital investments are available, the long-term performance reliability of DWSs depends on a robust workforce that can manage systems effectively and provide sustainable operation and maintenance (O&M). In this context, O&M is not merely about repairing pumps and replacing pipes—it is embedded within a complex socio-technical landscape that demands strong governance, skilled local workforce capacity, and coordination that extends beyond traditional disciplinary boundaries. Yet, this critical foundation is increasingly threatened by a nationwide workforce crisis, particularly acute in the decentralized wastewater sector.^2,5–8^ Workforce shortages—i.e., inadequate labor supply—limit the ability of limited-resource rural communities to fully leverage DWSs for broader economic development, missing opportunities to create stable local jobs, foster skill-building, and attract further investment.^9–11^ Still, the problem runs deeper than that of shortages alone. Workforce shortcomings—i.e., inadequate workforce capacity and technical preparedness—further weaken the sustainability and effectiveness of DWSs.

Despite these intertwined challenges, much of the scant literature^7,12,13^ on workforce development (WFD) in DWSs focuses almost exclusively on workforce shortages, often failing to consider the systemic shortcomings within the existing workforce that amplify these vulnerabilities. Even as national governmental agencies—including the U.S. Environmental Protection Agency (US-EPA)^5^ and the Congressional Research Service (CRS)^14^—acknowledge the escalating workforce crisis in the water and wastewater sectors, research and policy efforts continue to focus predominantly on shortages of installers and O&M workers. This narrow perspective is evident in regions, such as rural Alaska,^7,15^ Central Appalachia,^6^ and coastal North Carolina,^16^ where persistent workforce deficits—compounded by geographic, economic, and training-related constraints—leave water and wastewater infrastructure increasingly fragile. Here, we argue that effective DWS management in limited-resource rural communities must address the root causes behind both workforce shortages and shortcomings. These issues are deeply interconnected—increasing workforce numbers without addressing deficiencies in capacity-building mechanisms will not yield sustainable DWS solutions.

Beyond focusing narrowly on labor shortages and disregarding workforce shortcomings, current literature^13,17^ on WFD in DWSs also places disproportionate emphasis on the skilled trade workforce (e.g., installers, maintenance workers), often neglecting other career pathways needed for DWSs. According to the US-EPA’s Decentralized Wastewater Workforce Framework,^5^ building a reliable workforce for effective DWS management requires strengthening four major pathways: academic, regulatory, professional, and skilled trade. Challenges that affect stakeholders within a single pathway often have ripple effects across a broader workforce system. In many cases, these issues stem from misalignments or gaps between roles—dynamics that often go unnoticed when workforce challenges are examined within siloed institutional boundaries. This fragmented understanding obscures the root causes of workforce issues and leads to missed opportunities for effective intervention. Overlooking such interconnectedness among career pathways also limits our ability to identify more complex or intangible challenges that are embedded across institutional roles and relationships. Accordingly, this study proposes an integrated wastewater workforce approach that recognizes and leverages the existing affinities of each pathway to collaboratively address sector-wide problems. By drawing on the distinct strengths of each—and viewing them in relation to one another—this approach enables more practical, coordinated, and distributed responses to workforce challenges. From this perspective, identifying challenges that emerge at the intersection of the four major career pathways—referred hereafter to as “*cross-pathway challenges*”—can clarify how stakeholders’ responsibilities are shared, where silos form, and where targeted interventions are most needed.

The proposed cross-pathway approach seeks to answer the following question: In limited-resource rural communities, what are the core challenges driving wastewater workforce shortages and shortcomings when examined through the intersection of academic, regulatory, professional, and skilled trade career pathways? To answer this question, we draw on theoretical thematic analysis^18^ of 30 semistructured interviews with 35 stakeholders across the four career pathways. We demonstrate our approach on Alabama’s Black Belt—a rural region with longstanding wastewater infrastructure issues. Our analyses highlight how workforce dynamics emerge through the intersection of roles, expectations, and constraints unique to each pathway, offering insights that are often missed when examined in isolation. Further, our discussion addresses the recent US-EPA’s^5,19,20^ and CRS’s^14^ calls for strategies to advance water and wastewater WFD in rural communities. Although these agencies have recently emphasized this urgency, WFD in environmental-related fields has been recognized as a critical issue for over a decade.^21^ While this study is demonstrated on limited-resource rural communities in Alabama’s Black Belt, the cross-pathway approach used to examine workforce challenges can guide workforce planning in other limited-resource rural communities facing similar constraints, including rural Alaska, Appalachia, Texas Colonias, and Navajo Nation.^2^ Though each community operates within its own social and institutional context, this approach provides a practical entry point for developing WFD strategies that reflect the realities of DWSs while remaining adaptable across diverse geographies. Additionally, the proposed cross-pathway approach could inform efforts for addressing workforce challenges in other decentralized infrastructure sectors such as rural water, electricity, cellular, and solid waste management.

## 2 Material and Methods

### 2.1 US-EPA Decentralized Wastewater Workforce Framework

This study builds on the US-EPA’s Decentralized Wastewater Workforce Framework^5^ that emphasizes the need for a reliable workforce across four major career pathways: academic, regulatory, professional, and skilled trade. The regulatory pathway primarily includes public sector roles—such as regulators, policymakers, utility personnel, and oversight and enforcement personnel—including both public and private wastewater management entities. In some states, such as Alabama, utilities may be legally assigned regulatory responsibilities under state law; for instance, Alabama Administrative Code Rule 420-3-1-.16(3)^22^ requires wastewater management entities to establish procedures and guidelines in compliance with state rules, effectively positioning them within the regulatory sector. The skilled trades pathway encompasses private-sector workers, such as electricians, plumbers, and other tradespeople responsible for the installation and O&M of DWSs. The professional pathway includes engineers, consultants, and other professionals who design, assess, and optimize wastewater systems, often requiring higher education and advanced training in fields such as environmental engineering or soil and water science. Lastly, the academic pathway consists of professors and researchers who advance the field through teaching, workforce training, and applied research. Each of these pathways plays a critical role in achieving the sustainability and effectiveness of DWS management. Additional details on each career pathway are provided in Table S1 in the Supporting Information.

### 2.2 Study Site

Alabama’s Black Belt communities have long struggled from a lack of access to safely managed wastewater infrastructure—a consequence of decades of systemic underinvestment and exclusionary policies.^23^ The shrink-swell nature of the dense soil (vertisol clays) renders traditional DWSs (e.g., septic tanks and drain fields) ineffective due to hydraulic failure. Low population density and a lack of economic development further constrain the feasibility of advanced treatment technologies capable of adapting to these soil conditions. With 40% of residents living below the U.S. poverty line,^24^ many households were forced to rely on straight pipes that discharge raw sewage directly onto the ground surface, leading to significant environmental contamination and public health risks.^25^ For instance, a study of 2000 properties in Bibb County in Alabama’s Black Belt showed noticeable fecal material contamination on 50% of sites.^26^

Current research efforts on wastewater issues in Alabama’s Black Belt have focused on developing feasible treatment technologies^27,28^ and management models^29^ tailored to the region’s socio-technical constraints. However, like many other rural regions in the U.S., Alabama’s Black Belt faces persistent WFD challenges that limit the availability of a reliable workforce to support long-term O&M of DWSs.^30^ These workforce gaps undermine the effectiveness of such context-appropriate technical and managerial solutions. As such, Alabama’s Black Belt serves as a promising case to demonstrate our WFD cross-pathway approach. It sheds light on the significance of considering the intersection of four career pathways to provide more targeted, effective strategies for addressing both workforce shortages and shortcomings. Building a skilled localized workforce capable of maintaining DWSs and equipping local businesses with the necessary wastewater infrastructure are essential for supporting long-term economic development in Alabama’s Black Belt communities.

### 2.3 Data Collection

To identify the root causes behind wastewater workforce shortage and shortcomings in Alabama’s Black Belt, 30 semistructured interviews with 35 interviewees were conducted with a variety of community stakeholders (e.g., industry-based experts, regulators, community activists, local residents). Interviews began with general questions on wastewater challenges in limited-resource rural communities and then shifted to a flexible, respondent-driven discussion focused on each participant’s career pathway and their experiences with other pathways. This approach minimized interviewer influence and captured participants’ unique perspectives. Beyond the generalization of findings often pursued through quantitative analyses, qualitative research aims to uncover the richness and complexity of lived experiences within specific contexts. It prioritizes depth over breadth—aiming to understand the richness and complexity of lived experiences within a specific context.^31^ Our study focuses deliberately on the lived experiences of stakeholders within the decentralized wastewater workforce to uncover siloed dynamics and to codevelop recommendations grounded in their realities. Rather than relying on a single narrative, we triangulate across multiple perspectives—spanning four interconnected career pathways—to build a more systemic and transferable understanding. While the representativeness of the sample to the target population is not a metric typically sought in qualitative research, our cross-pathway structure broadens the scope of perspectives by drawing insights from regulatory, skilled trade, academic, and professional sectors.

Regarding sample size, the robustness of qualitative research is not determined by reaching a specific number of participants but rather by the achievement of theoretical saturation—the point at which no new themes or insights emerge from the interview data.^32^ In our case, saturation was reached after approximately 30 semistructured interviews. To further support this decision, we drew on established qualitative literature, which recognizes sample sizes of this scale as sufficient for achieving interpretive depth and advancing conceptual development.^33–35^ Indeed, studies across fields such as education, public health, communication, and policy analysis have relied on similarly sized samples to derive or refine theoretical insights and frameworks. Notably,^32^ our study adheres to the American Psychology Association (APA) guidelines for rigorous qualitative research by following the Consolidated Criteria for Reporting Qualitative Research (COREQ) matrix.^36^ This guideline ensures transparency in describing the research team, reflexibility, researcher-participant engagement, study design, and analysis; refer to Section 2 and Table S2 in the Supporting Information for further details.

The semistructured interviews—ranging between 40 and 96 min—were conducted between September 14th, 2022, and February 8th, 2023, via teleconferencing and were recorded, anonymized, and transcribed verbatim with the interviewees’ consent. We employed a combination of convenience sampling^37^ and snowball sampling^38^ to recruit participants. Initial respondents were identified through professional networks and ongoing collaborations (convenience sampling). We then leveraged interviewees’ knowledge of their communities to identify additional potential participants across the four career pathways (snowball sampling). To reduce potential bias and ensure broad stakeholder representation, we collected demographic data from interviewees, including job type, years of experience, income range, gender, ethnicity, and native language (see Table S3 in the Supporting Information). Capturing relevant demographics was essential for situating the knowledge produced in this study and providing a more accurate depiction of the broader workforce population studied.^39^ Most respondents were male (76%), and 66% had over 21 years of professional experience (Table S2). This gender distribution reflects the broader imbalance in the water and wastewater workforce, rather than a limitation of our study design. Institutional review board (IRB) approval was obtained from the University of South Alabama prior to data collection (IRB #: 22-224/1925043-2), which was conducted while the author was affiliated with the institution.

### 2.4 Data Analysis

Theoretical thematic analysis^18^—sometimes termed “deductive”—was performed on the interview data. This qualitative method enables the researcher to guide the analysis using a mature theoretical framework—in this case, the four career pathways defined by the US-EPA’s Decentralized Wastewater Workforce Framework:^5^ regulatory, skilled trade, academic, and professional. Originating from the theoretical framework, this analytical approach allows deeper insights to emerge based on the qualitative data, facilitating the identification of context-specific challenges in Alabama’s Black Belt. Notably, the coding unit was defined as a complete idea expressed by the interviewee, with each unit being eligible for multiple codes simultaneously if thematically relevant. Our coding and analysis unfolded in three critical phases, each playing a fundamental role in structuring the study’s findings.

In Phase 1, we created thematic codes under each of the four career pathways based on excerpts describing challenges faced, identifying what we term “pathway-specific challenges”. These challenges are unique to an individual career pathway within the decentralized wastewater workforce, arising from the specific roles, responsibilities, and constraints of that pathway. For example, consider the following excerpt:

*“A lot of times you get kids that [taking tests is] just not their thing. You put a motor in front of them; they can take it apart and put it back together again. But you want them to read, write, and do math; [they will struggle]. So, a lot of the tutoring [needed] is in math.”*

This excerpt was coded as both a skilled trade challenge (difficulty for skilled trade workers to pass certification exams) and an academic challenge (misalignment between provided training and required certification testing).

In Phase 2, we shift the focus from pathway-specific challenges to how pathways intersect with each other. To do that, we created co-occurrence tables (see Section 3.2) to identify (1) instances where stakeholders in one pathway reported challenges related to other pathways and (2) individual excerpts that simultaneously highlighted challenges spanning multiple pathways. For example, the excerpt noted above is one of the many situations that created a link between pathway-specific challenges across two pathways—here, academic and skilled trade—increasing the observed co-occurrence between them. Notably, pairwise co-occurrence scores helped uncover shared concerns between two pathways. However, by themselves, these counts did not reveal whether these links represented isolated overlaps or symptoms of deeper system-wide challenges.

In Phase 3, we moved beyond pairwise overlaps to examine whether these interconnected issues pointed to deeper systemic cross-pathway failures. To illustrate: if in Phase 1 we identified pathway-specific challenges A, B, and C and in Phase 2 we observed that A was linked to B and B to C, Phase 3 asked whether these links indicated a broader structural problem also encompassing A and C. Because this assessment was a qualitative—not quantitative—process, we could not simply assume that such indirect connections (like A to C) existed based on co-occurrence data alone. Instead, to determine whether these overlaps constituted genuine cross-pathway challenges rather than coincidental intersections, we conducted an iterative comparison of co-coded excerpts and assessment of contextual relevance and triangulated our interpretations with field-specific knowledge and literature^40–42^ to robustly substantiate these connections. This approach is further illustrated by a second example excerpt:

*“There’s no operator training or test for decentralized [wastewater treatment systems] in Alabama...So, they [individuals seeking to become operators] have to take the same test as this other guy who works at the central plant—totally different operation.”*

Viewed alongside the earlier excerpt, these statements showed how regulatory standards, academic curricula, and skilled trade certification gaps were not isolated problems but interconnected components of a broader cross-pathway challenge rooted in siloed institutional practices. This specific connection is elaborated in Section 4.2 as the cross-pathway challenge “Siloed Academic, Regulatory, and Professional Pathways Hindering Skilled Trade Development”. While these examples simplify the logic for clarity, building these connections to identify cross-pathway challenges becomes far more intricate when factoring in the plethora of pathway-specific challenges and the sheer volume of contextual data generated by interview transcripts due to their complexity and interconnectivity. The structured cross-pathway approach developed here provides a structured way to navigate that complexity, allowing for rigorous and replicable identification of cross-pathway challenges.

Throughout the analysis phases, interviews were coded using NVivo 15 software (version 15.1.2).^43^ Codes were iteratively refined to reflect subthemes or indicate the emergence of new themes. To ensure reliability, a first researcher coded all 30 interviews twice, followed by an intercoder reliability check with a second researcher to reduce inherent bias. Coding discrepancies were reviewed and resolved through consensus meetings. Intercoder agreement was assessed using Mezzich’s Kappa calculated on 10% of the data set, yielding a score of 0.70—considered solid for qualitative research.^44^

### 2.5 Study Rigor and Limitations

Out of the 32 rigorous qualitative research reporting criteria in the COREQ matrix,^36^ 27 were met and 5 were deemed irrelevant (see Table S2 in the Supporting Information). Furthermore, rigor in qualitative research is characterized by two main components: validity and reliability.^40^ In addition to meeting the intercoder reliability requirement, we addressed validity through three key components: authenticity, plausibility, and criticality;^40^ refer to Section 2 in the SI for further details.

While we thrive to enhance study rigor, some limitations must be acknowledged. Although fewer interviewees represented the academic pathway, the study’s cross-pathway design enabled the identification of robust academic-related challenges. These emerged not only from academic participants but also from professionals, regulators, and skilled trade workers who interact with or are shaped by the academic sector. Thematic saturation was reached, although future research may benefit from additional academic perspectives to deepen cross-pathway insights. Additionally, two limitations stem from the US-EPA’s Decentralized Wastewater Workforce Framework.^5^ First, the regulatory pathway includes a wide spectrum of roles—from inspectors to grant writers and policymakers—which may obscure nuances. To address this, we specify each interviewee’s role when quoting them. Second, the framework excludes community members despite their critical role in workforce development. We addressed this by including interviews with residents and activists to reflect their overlooked involvement. Future adaptations of the framework should recognize communities as distinct stakeholders and disaggregate the regulatory pathway to better represent its internal diversity.

Even though this study is demonstrated on Alabama’s Black Belt, the cross-pathway approach is adaptable to other limited-resource rural areas—such as rural Appalachia, Texas Colonias, Navajo Nation, and tribal lands—facing similar workforce shortages, governance fragmentation, and infrastructure vulnerabilities. Still, we acknowledge that the specific social context—shaped by local governance, culture, environment, and policy—will affect both the nature of workforce challenges and the viability of potential solutions.

## 3 Results

### 3.1 Pathway-Specific Challenges in the Decentralized Wastewater Workforce

Table S4 in the Supporting Information presents selected pathway-specific challenges identified in Phase 1, along with corresponding descriptions and illustrative excerpts. For instance, within the academic pathway, training programs fail to prepare candidates to pass their certification examinations. The professional pathway faces difficulties in adapting to small-scale treatment technologies and incorporating community needs into system designs. The regulatory pathway is hindered by ineffective stakeholder communication, inconsistent enforcement, and rigid regulations that stifle innovation. Meanwhile, the skilled trade pathway is burdened by workforce retention issues due to limited apprenticeship opportunities and inadequate compensation. It is important to note that while the ultimate objective of this study is to illuminate how examining the interconnectedness across these challenges reveals deeper insights—culminating in the identification of cross-pathway challenges—understanding the pathway-specific challenges first laid the necessary foundation for detecting where and how systemic breakdowns occur across pathways.

### 3.2 Initial Indicators of Cross-Pathway Challenges

Table 1 illustrates how pathway-specific challenges were reported across different stakeholder groups. Each row represents the group reporting the challenges, and each column corresponds to the career pathway in which those challenges were observed. For example, the first row indicates how frequently academic stakeholders reported challenges in academic, professional, regulatory, and skilled trade pathways. This structure allows us to assess how each group perceives workforce shortcomings and identify which pathways are most frequently associated with the reported challenges.

**Table 1:** Distribution of Pathway-Specific Challenges Across Wastewater Workforce Career Pathways^a^

Pathway	Academic	Professional	Regulatory	Skilled Trade	Total^b^
Academic	8 (22%)	5 (14%)	15 (42%)	8 (22%)	36 (100%)
Professional	48 (15%)	70 (22%)	121 (39%)	73 (23%)	312 (100%)
Regulatory	42 (19%)	25 (11%)	102 (46%)	54 (24%)	223 (100%)
Skilled Trade	35 (30%)	17 (15%)	32 (28%)	32 (28%)	116 (100%)

a Shown as count (percentage). Note: The percentages sum to 100% across each row.

b Total column indicates the total number of challenges reported about each career pathway, regardless of the stakeholder group reporting them.

A key observation from Table 1 is that regulatory pathway challenges were frequently reported not only by other stakeholder groups but also by regulatory stakeholders themselves. Specifically, 46% of the concerns raised by regulatory respondents pertained to their own pathway. Regulatory challenges were also the most frequently mentioned by academics (42%) and professionals (39%), suggesting this pathway may be a focal point of sector-wide workforce issues. In contrast, skilled trade respondents most often reported challenges in the academic pathway (30%).

Table 2 shifts the analytical focus toward identifying when a single excerpt was coded as relevant to challenges in multiple career pathways. In this structure, the diagonal cells indicate the total number of excerpts coded to each individual pathway, while the off-diagonal cells capture the number and percentage of those excerpts that were simultaneously coded to another pathway’s challenges. For instance, the first row shows that 15 (10%) excerpts coded under academic challenges also contributed to professional challenges, 37 (25%) contributed to regulatory challenges, and 26 (17%) contributed to skilled trade challenges. When one looks at both tables, Table 1 shows that 30% of skilled trade stakeholders reported concerns about the academic pathway and Table 2 shows a 14% overlap in excerpts coded to both academic and skilled trade pathways, suggesting a shared underlying concern. These patterns informed our deeper interpretive analysis, guiding us to investigate whether such overlaps reflected broader cross-pathway challenges.

**Table 2:** Co-occurrence of Coded Excerpts Amongst Pathway-Specific Challenges^a^

Pathway-Specific Challenge	Academic	Professional	Regulatory	Skilled Trade
Academic	150 (100%)	15 (10%)	37 (25%)	26 (17%)
Professional	15 (12%)	128 (100%)	47 (37%)	20 (16%)
Regulatory	37 (10%)	47 (13%)	366 (100%)	34 (9%)
Skilled Trade	26 (14%)	20 (10%)	34 (18%)	191 (100%)

a Shown as count (percentage). Note: Percentages indicate how often challenges from each row co-occurred with the total number of excerpts coded for the pathway listed in each column (i.e., diagonal cell).

## 4 Discussion: Core Cross-Pathway Challenges in the Decentralized Wastewater Workforce

Six cross-pathway challenges emerged from the analysis (see Table S5 in the Supporting Information), each reflecting a systemic issue that cuts across multiple career pathways.

### 4.1 Lack of Structural Support for O&M Entrepreneurial Endeavors

Rural entrepreneurship is defined as economic endeavors based on localized resources and the uniqueness of a geographical area.^45^ This form of entrepreneurship, unique to rural locations, can be a lifeline for limited-resource communities by addressing unique local needs and creating opportunities for economic growth. In Alabama’s Black Belt, the lack of competition in the O&M sector of DWSs presents a unique opportunity for entrepreneurial ventures. The persistent workforce shortages and infrastructure challenges indicate an unmet demand for sustainable O&M solutions. If effective competition existed in this space, these market-wide issues would likely be less pronounced, as market-driven incentives would encourage WFD, service efficiency, and innovation. The existence of these challenges underscores the need for entrepreneurship to fill critical gaps in service provision and workforce capacity.

However, localized challenges are impeding the development of management entities focused on the O&M of DWSs in Alabama’s Black Belt. In the professional career pathway, for example, the blame is pointed toward the perceived innate lack of profitability in the wastewater sector. One of the engineers interviewed portrays the governing mentality when it comes to entrepreneurial activities in the wastewater sector: *“Pretty much everybody feels like sewer is not profitable at all. Sewer is where you just run into all sorts of financial mess because it does not make money. It just doesn’t. And why would you want to get involved with something that doesn’t make money?*” The skilled trade career pathway agrees with the professionals when it comes to the potential for career development and profitability of the field. An operator experienced with the finances of his company describes the situation “*Wastewater is not a profitable business. It’s a break even. [...] It’s rare that sewage is going to pay for itself, but if you can get close to break even, it’s the best thing that can happen.”* Researchers in the field of entrepreneurship propose multiple solutions for business endeavors in limited-resource areas. They suggest that emerging companies do not need to create a new market to generate revenue, but they can instead leverage existing capabilities, form strategic partnerships, and optimize resource configurations to create sustainable business models.^46^ This approach is particularly relevant for decentralized wastewater O&M, where the service and infrastructure needs already exist, but the absence of competition and perceived lack of profitability deter private investment. Businesses in wastewater management could mitigate financial risk by aligning with public entities, nonprofit organizations, and development agencies to access funding, reduce initial capital costs, and improve service delivery efficiency.^46^ Additionally, existing O&M models could be scaled rather than reinvented, allowing future businesses to integrate their experience in the decentralized wastewater industry into the community while maximizing operational efficiency. Emergent business could also leverage regionalized O&M models which may ease some of the financial burden by broadening their customer base and thereby increasing revenue.^47^

While these strategic approaches present potential solutions for addressing the challenges in wastewater workforce, their implementation is far from straightforward without the cooperation of the regulatory sector.^48^ Current regulations are perceived as too prescriptive, driving out innovation in wastewater management. As a private utility owner describes it, *“The biggest problem with the regulatory business [...] is that regulators like to have a book with all the pages. It says on this page that if you got this condition, [then] this is what you have to do. That’s called prescriptive regulation. Every problem has a prescription to fix it. [...] We have been fighting for the last three decades to get regulations changed from prescriptive to performance based.”* Prescriptive regulations aim to enforce a strict methodology or technology and prevent individual companies from innovating and introducing new solutions to the market. While this approach could be beneficial in more uniform urban regions and in more industrialized sectors, the specificity of limited-resource rural communities and the diversity of wastewater technologies require more flexible regulations while maintaining the same quality standards. Therefore, future policy makers should focus on establishing performance-based regulations which have been shown to be more politically accepted by prospective companies.^49^

Specifically, the Alabama Administrative Code Chapter 420-3-1 ^22^ reveals multiple instances of highly prescriptive criteria. A notable example is Rule 420-3-1-.53, which mandates gravel field sizing for effluent disposal based solely on soil classification and number of bedrooms—offering little flexibility for alternative system designs and disregarding actual system performance or treatment outcomes. Rule 420-3-1-.54 provides limited allowances for gravel field size reductions (e.g., a fixed 33% area reduction if gravel depth is increased or if advanced treatment is used) but prohibits combining these reductions and offers no pathway for site-specific performance demonstrations. While these provisions appear flexible on paper, they remain restrictive in practice and hinder innovation. To foster more effective and adaptable regulation, policymakers should prioritize predictability by establishing clear, consistent expectations over time that allow stakeholders to plan and invest with confidence.^49^ Predictability can be achieved by establishing clear long-term environmental goals and incorporating cost thresholds, flexible compliance pathways, and alignment with existing regulations to minimize inefficiencies and avoid unintended conflicts. In the gravel bed sizing example, a performance-based approach would allow engineers to propose designs that demonstrate equivalent or improved pollutant removal and infiltration outcomes through site-specific testing. Such an approach would shift the regulatory focus from rigid design specifications to measurable environmental performance. The Florida Administrative Rules 62-6.025 through 62-6.0295 ^50^ provide a useful precedent. Performance-Based Treatment System Policy under these rules allows for alternative onsite system designs, provided that they meet effluent quality standards verified through sampling and monitoring. Operators must obtain special permits and demonstrate compliance through field testing—ensuring accountability while enabling design flexibility. Alabama regulators could adopt similar frameworks by developing standardized verification protocols, establishing pilot-testing tracks, and implementing expedited review procedures for validated designs. Such a framework would encourage innovation, support entrepreneurship, create more room for competition, and enhance the long-term sustainability of DWSs.

Surprisingly, an executive-level regulator asserted that their agency employs a performance-based policy approach: *“Well, our regulatory responsibility is to make sure that whatever [water] they’re discharging meets quality standards. It’s up to them [design engineers] to determine how they achieve that water quality.”* This statement reveals a disconnect between regulatory intent and practice—particularly striking given that design engineers working in the same jurisdiction described the regulations as highly prescriptive and inflexible. This misalignment suggests that regulators may not be actively engaging with the very professionals they oversee, undermining the effectiveness and practicality of the regulatory framework. Effective regulation requires continuous communication and collaboration among stakeholder groups. Yet, the absence of such engagement creates a regulatory environment perceived by design engineers and skilled trade workers as “high-risk, low reward”—a sentiment echoed across professional, regulatory, and skilled trade career pathways (see Table S5 in the Supporting Information). This perception discourages private-sector participation in Alabama’s Black Belt, stifling local entrepreneurship and one of the key drivers of WFD—that is, open market competition.

The applicability of these findings could be extended beyond the decentralized wastewater industry. Similar regulatory and financial barriers are present in other rural infrastructure sectors—such as healthcare, transportation, and energy—where rigid regulations and financial risk hinder local investment and innovation. Adopting more genuinely performance-based regulatory frameworks could enable rural entrepreneurship, thereby fostering self-reliant local enterprises, and creating new employment opportunities in limited-resource rural communities.

### 4.2 Siloed Academic, Regulatory, and Professional Pathways Hindering Skilled Trade Development

Ideally, actors within an infrastructure system should maintain open channel communication with one another to enable effective problem-solving, conflict prevention, and responsive decision-making. In practice, however, many agencies often struggle with the effective implementation of these communication channels leading to siloed entities.^51^ A prime example of this shortcoming is the disconnect within the regulatory-academic-professional triangle. Academics are typically responsible for designing training materials for prospective skilled trade workers and shaping engineering programs that prepare students to enter a professional career pathway. In theory, these programs are intended to equip students with the technical skills and background to meet employer expectations.^52^ Unfortunately, in practice, the siloed nature of academic and professional pathways resulted in a misalignment between education and workforce needs—especially in the decentralized wastewater management sector.^53^ This misalignment and its consequences are portrayed by an engineer working for one of the few companies in Alabama’s Black Belt managing both installation and O&M of DWSs: *“When we’re hiring engineers, we’re measuring their aptitude. Then, we’re spending a year training them ourselves because they don’t come out of school with any skills relevant to what we and the industry needs. We have invested a lot of time and money in some of them, and [then] they leave to much bigger jobs, beyond [company name] because suddenly, they have this training.”* Most engineering programs continue to prioritize centralized wastewater treatment systems that are designed for urban contexts. As a result, engineers often graduate with little to no exposure to DWSs, which can skew their design approach toward larger, more complex, and often overengineered solutions—frequently omitting DWSs entirely. This gap in training may lead rural communities to adopt technologies that are misaligned with their needs and exceed the operational capacity of their local governments simply because the engineer lacks familiarity with DWSs. A technical assistance provider described the implications of this disconnect: *“People should make realistic decisions about the type of system that goes into these small communities. Instead, they are paid for a Cadillac system, one that the community doesn’t have the resources to staff or maintain with someone capable of running that top-notch, cutting-edge treatment system.”* This lack of exposure to DWSs within engineering education highlights the need for stronger collaboration between academic institutions and professionals to ensure that curricula are responsive to sector-specific workforce demands. Notably, a rare decentralized wastewater treatment course piloted in one institution demonstrated high student engagement and interest rates and emphasized the value of integrating technical knowledge, policy understanding, and real-world applications.^54,55^ The consequences of this disconnect between academics and professionals also extend downstream to the skilled trade workers. As one senior operator explained: *“I think, last year, there was twice as many people leaving the business [wastewater industry] as getting into it. In Alabama, the problem they [management entities] are having now is [policymakers] have made it so hard to become one [a licensed operator]. It’s gotten to the point to where they require you to know so much knowledge about stuff that has nothing to do with [the actual job].”* This statement underscores how poor alignment across career pathways can distort licensing requirements, deter entry into the workforce, and eventually undermine workforce retention in a sector that can ill afford further attrition.

A compelling example of academic-professional collaboration is the 2005 initiative by Chicago policymakers to align workforce training with employer needs in the manufacturing and service industries.^56^ The efforts helped companies identify skill gaps and provided consultations on process improvements and training material development through designated workforce centers. While the initiative effectively supported employer-driven training efforts, it struggled to balance the needs of employers and workers. By prioritizing employer needs, the program raised concerns about neglecting employees’ personal growth and career development.^56^ The overreliance on the professional sector in the training of the future workforce could put the individual worker at risk of predatory policies. A similar pattern was observed in a 2008 initiative in England that sought to bridge university-level workforce development with the professional sector. Although the collaboration initially fostered industry-relevant training, it leaned too heavily toward profit and growth, leading to decline in educational standards.^57^ These examples highlight a key risk: when training efforts are dominated by the professional sector, the individual worker may be exposed to predatory practices driven by corporations’ natural striving for profit. This imbalance underscores the critical role of the regulatory pathway in WFD. Regulators can intervene to mediate between the academic sector’s emphasis on traditional research practices and the professional sector’s market-oriented priorities. Effective regulatory involvement can maintain high quality standards, break down the silos around curricula development and training material, and ensure alignment with workforce needs, as defined by employers. Germany offers a promising model of university-wide collaboration with employers from diverse sectors. There, industry representatives serve on university governing boards and advisory committees, where they provide structured feedback on curriculum design and skill requirements.^58^ However, unlike purely employer-driven models that prioritize profit over educational depth, Germany’s regulatory bodies enforce minimum quality standards to ensure that vocational training does not devolve into profit-driven predatory practices. Such a system ensures that students gain both theoretical knowledge and practical skills, preparing them for the dynamic labor market without sacrificing educational rigor. By applying this model to the wastewater management sector, regulatory entities could mandate codeveloped training programs between universities and industry professionals, supported by an oversight framework. Such a framework would guarantee technical proficiency, research integration, and long-term career development while preventing short-term, profit-based job placement. Ultimately, sustained collaboration across the academic, professional, regulatory, and skilled trade career pathways (see Table S5 in the Supporting Information) is essential for workforce training programs that are academically rigorous, industry-relevant, and responsive to shifting workforce demands. It is this balance that ensures the long-term sustainability of the decentralized wastewater workforce.

### 4.3 Cultural Barriers to Development Efforts: Breaking through Professional, Regulatory, and Social Resistance

Cultural resistance is a persistent challenge in development projects, particularly in low-resource rural communities, where past failures and external interventions have fostered deep-seated skepticism. While technical solutions and policy reforms are often proposed to address workforce shortages and infrastructure gaps, these efforts frequently overlook the cultural dimension specific to each community and attempt to impose a one-size-fits-all solution.^59,60^ This approach represents a critical workforce shortcoming as the workers in charge of designing and implementing these development efforts fail to account for the unique social, economic, and historical contexts that shape workforce challenges and solutions in different regions. In Alabama’s Black Belt and other rural regions, social resistance to professional, regulatory, and academic initiatives is not a mere community-specific trend. Rather, it is a historically entrenched phenomenon shaped by decades of imposed policies, failed interventions, and exclusion from decision-making structures. Multiple community activists reported significant pushbacks from the community during the implementation phase of funded development efforts, for example, *“when they didn’t get community support on a project, and when the community found out, they killed the project because they didn’t feel included. Even if it was a great project for the entire community, it was going to have great economic impact, but it was the fact that they weren’t engaged in the process. Legally they had covered their bases as it relates to public notice processes, but it’s the fact that they didn’t garner enough community support.”* Such community’s reaction does not represent a blatant opposition to progress but rather an expression of the need to be included in the planning and decision-making of infrastructure development efforts.

When initiatives are imposed—whether by external agencies or internal decision-makers who fail to consult the broader community—the result is often distrust and rejection, regardless of the project’s potential benefits, as evidenced by the previously referenced interviewee. Development initiatives that do not involve the community in the preliminary planning stages are perceived as top-down interventions, reinforcing skepticism rather than fostering the residents’ desire to join the workforce and have long-term commitment to the project. Smaller rural communities are more resistant to imposed changes due to their tighter social fabric, dependence on localized economies, and historical experiences. Unlike urban environments, where large populations and continuous infrastructure development dilute the intensity of perceived change, rural communities operate within highly interconnected social networks where even small disruptions can have outsized economic, cultural, and political consequences.^61^ Additionally, rural economies tend to be more fragile, with fewer alternative employment opportunities and a greater reliance on land, water, and infrastructure—making any disruption more likely to impact daily life. As a result, externally driven efforts—especially those perceived as disruptive, untested, or misaligned with local priorities—are met with stronger resistance than they might be in urban settings, where change is a more routine aspect of development. This resistance within the community goes beyond rejecting imposed initiatives; it can also manifest in workforce disengagement. When residents lack trust or belief in a project, they are unlikely to view employment within it as a viable career path. Without strong community buy-in, the project risks being perceived as external or unwelcome, leading locals to withdraw not only their support but also their participation as potential workers. This dynamic can jeopardize both implementation and long-term workforce stability, reinforcing perceptions of the project as unreliable or unsustainable.

Community buy-in can also be directly linked to some of the professional career pathway’s struggles. Multiple interviewees referenced tensions among residents, professionals, and skilled trade workers, particularly as the latter are often the visible face of infrastructure development efforts and may be perceived as agents of disturbance. For example, a *“contractor had dug up the right of way space by this business owner”*, leading to frustration and complaints. In this case, a simple phone call between the contractor and the business owner resolved the issue and de-escalated tensions. These situations underscore a key workforce issue in the professional pathway: technical competence alone is insufficient for successful project implementation. Professionals in the wastewater sector must also develop skills in stakeholder engagement, community relations, and public communication to navigate the complex social landscape of rural wastewater development efforts. This issue is further reinforced by a community activist who challenged the flawed assumption that professional expertise should override local knowledge: *“No one had a discussion with the community to teach them, train them, or inform them on how they need to handle things. In the town meetings, it’s more or less a dictatorship telling [the residents], ‘This is what we [the utility or regulatory entity] are going to do, because this benefits us’. Not giving [the residents] the problem, explaining the issue, and listening to what [the residents] have to offer to understand their position. I have often said that just because they are professionals, doesn’t mean no one else knows what they know.”* This analysis reflects one of the core tensions within the wastewater workforce: professionals often underestimate the practical knowledge and lived experiences of local residents and community members. This mindset may stem from a broader lack of awareness among professionals and decision-makers about the importance of community engagement in rural areas. A lawyer with tight connection to the community emphasized this point: *“I think that there’s this perception that public participation and community input, these people are just a pain in the rear end. And we ‘know best and why should we listen to them?’ And I think that’s a huge mistake.”* This sentiment has also been echoed by an engineer who explicitly acknowledged that *“they [the community] do add hardship to the process. They can wreck ideas.”* Together, these insights reveal how dismissive attitudes toward community input not only alienate residents but also undermine the long-term sustainability of infrastructure initiatives in rural communities.

The exclusion of the community voices is not limited to the professional and regulatory career pathways; it is also evident within the academic career pathway. As one community activist noted, *“[When the initiative is] from a university, the community, culturally, thinks it’s an experiment. They think ‘Okay, we don’t want to be your Guinea pigs, your test subjects on that.’ When it comes from a university, it automatically creates a sense of pause.”* Although recent academic efforts have increasingly emphasized community-based approaches, their practical implementation often falls short. Many academic-led initiatives still fail to engage local residents effectively throughout all stages—from problem definition and project design to implementation and evaluation. This incomplete inclusion reinforces the perception that academic involvement is extractive rather than collaborative. While some progress has been made in acknowledging the importance of community engagement, academic efforts often remain passive, relying on consultation. To shift this dynamic, academics must work more closely with communities on the ground, codeveloping and cocreating solutions that reflect local priorities and knowledge rather than imposing externally driven frameworks.^62,63^ Beyond developing community engagement practices and policies, the academic sector can play a critical role by initiating education initiatives that foster long-term cultural and technical literacy around DWSs. Early K–12 interventions, for example, can gradually dismantle misconceptions about wastewater work and encourage environmental responsibility—outcomes demonstrated by school-based water, sanitation, and hygiene (WASH) programs that successfully improved knowledge transfer within households and across communities.^64^ Complementary community education strategies—such as citizen science and participatory water-quality monitoring—further create opportunities for residents to coproduce data with regulators, thereby strengthening transparency, trust, and local ownership of infrastructure solutions.^65^ Collectively, these multilevel educational initiatives help bridge knowledge disparities between residents and regulatory bodies, ultimately reinforcing collaboration and long-term workforce sustainability in decentralized wastewater management. These analyses highlight that this pattern of community exclusion from decision making— whether stemming from systemic policy gaps or limited community awareness —is a pervasive issue across all four decentralized wastewater career pathways, as illustrated in Table S5 in the Supporting Information.

To address this persistent exclusion, the four pathways must adopt systemic policies for community engagement, as the minimum standards imposed by current policies fail to address residents’ needs and ensure effective resource allocation. In accordance with the Alabama Administrative Code for Public Notification,^66^ *“the agency shall make its best efforts to notify the public of the proposed rule. [···] The agency shall post the text of the rule the agency proposes to adopt, amend, or repeal on its website.”* However, this requirement is limited to passive notification; it does not mandate the inclusion of community members in the design, deliberation, or revision of the rule itself. The public is informed after decisions are made and not consulted during the design process, which limits the potential for regulatory rules to be responsive to local needs. Without meaningful engagement, infrastructure development efforts risk inefficiency, community resistance, and missed opportunities for sustainable solutions. Such meaningful engagement policies are implemented in New York City where community participation is legally institutionalized. The Civic Engagement Commission, established under Chapter 76 of the New York City Charter,^67^ implements participatory budgeting in which residents collaboratively propose, develop, and vote on public projects. The process spans several months, starting with community brainstorming sessions, followed by proposal development with city agency support, districtwide voting, and final evaluation and planning—ensuring that infrastructure and budgetary decisions reflect the direct input of those they affect. Bakchan et al.^29^ provided evidence showing that neglecting the social aspect in infrastructure management can directly and severely affect the technical performance of infrastructure systems in urban contexts. The current study extends these insights to limited-resource rural communities, further emphasizing that the exclusion of community voices can have equally—if not more—severe consequences on the effectiveness and sustainability of technical infrastructure services. As such, a more structured, proactive approach—such as mandatory stakeholder consultations and participatory decision-making like the initiative created by the New York Civic Engagement Commission^67^—is essential to foster trust, improve project outcomes, and maximize the long-term impact of infrastructure investments.

### 4.4 Generational Divide in Gen Z’s Workforce Expectations and Realities

As Gen Z (people born between 1997 and 2012) start to join the workforce, senior managers from older generations (e.g., Millennials 1981–1996, Gen X 1965–1980, Boomers 1946–1964) start to face challenges that they have not experienced before. Gen Z, often described as “digital natives”, have been reported to show contrasting differences in priorities with their older superiors.^69^ An operator describes his experience with younger workers: “*Well, it depends on the age group. Under 30, they’ll probably go somewhere with higher pay. Over 30, then they’re more concerned about benefits [···]. The 30 and under aren’t too worried about needing health insurance now.”* Workforce researchers report conflicting evidence regarding Gen Z’s job priorities. Some studies^57,70^ show that preference is given toward nonmonetary benefits, such as social aspects related to team spirit, working atmosphere, and job-life integration. In contrast, other studies^58,71^ emphasize a prioritization of monetary compensation. In rural communities’ with deeply rooted historical and cultural contexts—such as Alabama’s Black Belt—socio-cultural factors may play an even greater influence on local Gen Z’s employment decisions.^72^ Our findings reveal a distinct generational gap between Gen Z and their older employers or managers, highlighting the need for community-specific research to better understand the priorities and workplace expectations of the emerging workforce. Creating an environment that aligns with these expectations is essential for retaining young workers and harnessing the skills unique to their generation. This tension is exemplified in one interviewee’s concern: *“Once you get them certified, the neighboring community who has more dollars might snatch them away from you. You spent all the money training them, now they have higher qualifications, and they can go to the next major town to get more money.”* While such behavior may seem disloyal or short-sighted to Boomer or Gen X employers, job-hopping is far more normalized among Gen Z,^73^ who often seek improved compensation or workplace environments that better align with their values. Despite these shifting expectations, utilities have been slow to adapt. In numerous cases, the barrier is not simply a lack of resources but a lack of strategic foresight. Many smaller utilities lack even the managerial capacity to implement the most basic retention and succession plans,^74^ leaving workers in environments where there are few, if any, organizational mechanisms to address dissatisfaction, burnout, or professional development needs—factors widely reported as major retention inhibitors.^75^ Before adopting complex or innovative retention strategies, utilities must first establish a foundational human infrastructure. Without this baseline, even well-designed programs will fail to take root. For this reason, building human resource capacity—through dedicated personnel responsible for workforce planning, performance evaluation, and professional development—is not merely an administrative improvement but a core component of any long-term retention strategy. Utilities can begin by dedicating personnel to human resource management functions—responsible for workforce planning, performance evaluation, and professional development. Academic institutions can complement these efforts by training utility leaders to value human infrastructure management as equally critical to system sustainability as to technical operations.

Moreover, the interview data suggest that employers recognize the importance of engaging the future wastewater workforce at an earlier stage—often during high school or community college—to spark interest in the field. For instance, one interviewee noted, *“We’ve got to start reaching out to people in high school or even community college, to get people interested in the field.”* However, despite their extensive experience, none of the interviewees acknowledged the need for a two-way dialogue with younger populations to identify the challenges preventing them from joining the workforce. Some of these challenges could be purely technical or logistical in nature. For example, in the transportation workforce sector, Gen Z workers have reported frustration with outdated technologies—such as desk phones and fax machines—that remain common in government offices and public utilities.^76^ While upgrading office equipment might appear to be a frivolous expenditure—given that it is a tangible and easily identifiable change—it symbolizes a broader resistance to modernizing work environments in ways that resonate with younger employees. If institutions resist updating even basic tools, they are unlikely to adopt deeper structural reforms in workplace culture, policies, communication practices, and technological platforms that make infrastructure careers more attractive to Gen Z. In the decentralized wastewater sector, the adoption of AI-driven cyberinfrastructure—such as remote monitoring, predictive maintenance, and automated operations—offers utilities the potential to manage DWSs more efficiently and reliably.^77^ Beyond operational gains, such technologies may help render the sector more appealing to technology-oriented Gen Z professionals by signaling that the industry is responsive to contemporary workforce trends.^78^ It is important to acknowledge that, in some sectors, predictive systems and automation have enabled leaner staffing models—such as in industrial predictive maintenance contexts, where machine learning models allow smaller teams to monitor and act proactively.^79^ However, the decentralized wastewater sector already confronts a documented workforce shortage.^5,20^ In this context, cyberinfrastructure does not displace workers but instead enables retention and upskilling by allowing utilities to concentrate higher compensation and more meaningful tasks on essential staff, while also creating clearer career growth pathways tied to cyberinfrastructure (e.g., from field operator to systems/data analyst to supervisor). This strategy is consistent with findings from both academic studies^80,81^ and reports from consulting firms^82,83^ that a lack of perceived career advancement is among the top drivers of voluntary turnover. By embedding formal promotion ladders, role transitions, and upskilling opportunities within digital transformation plans, utilities can link technological modernization with workforce retention goals. This broader resistance to modernization extends beyond outdated technologies and into communication practices, which are increasingly important for attracting and retaining a younger professional. Unlike private firms in urban settings, government agencies and utilities often underutilize social media and, when they do engage, their messaging fails to resonate with younger audiences.^76^ In contrast, modernized offices have increasingly adopted simple yet effective communication tools,^84^ such as Slack,^85^ Basecamp,^86^ and Microsoft Teams,^87^ which promote collaboration, foster real-time engagement,^84^ and improve workforce training, retention and efficiency.^88^ These platforms promote transparent communication, reducing the inefficiencies inherent in traditional hierarchical communication channels. Yet, many public-sector workplaces continue to rely on slow, opaque methods of information dissemination, further alienating Gen Z employees accustomed to instant, digital interaction. This disconnect is evident in the experience of a local grant writer who expressed frustration with the government’s internal communication inefficiencies: *“It is hard to reach everybody. Sometimes I feel like the message just doesn’t get passed along [effectively]. I can tell you that I forward stuff to our elected officials all the time. Whether they read it or not? I don’t know. Whether they share it with their staff? I don’t know. Whether they pass it on to anybody that it would be useful to, that they know more directly than me? I’m doubtful, I really am.”* This communication breakdown reinforces the perception that government agencies are out of touch with the expectations and needs of the emerging workforce. Bridging this generational divide requires mutual adaptation: younger employees must learn to navigate existing workplace structures, while employers must modernize their tools and mindsets. Investing in contemporary communication infrastructure, reevaluating rigid workplace policies, and creating open feedback mechanisms can help align intergenerational expectations. Without such efforts, the disconnect will deepen, making it increasingly difficult for public and private wastewater employers to attract and retain the next generation of workers.

As discussed in Section 4.3, these findings further reinforce the need for stakeholders to cocreate WFD strategies in partnership with the community. As illustrated in Table S5, the four career pathways must work collaboratively to leverage the vast variety of skill sets available across sectors. For example, professionals can draw on the expertise of academics who specialize in community engagement to improve communication with the emerging applicant pool—namely, Gen Z community members—and cocreate a work environment that values both senior employees and the incoming workforce. This type of generational disconnect is not unprecedented. In fact, a similar shift occurred in the early 2000s, when a new cohort of workers—fluent in digital technologies—entered workplaces still reliant on outdated systems.^89^ A comparable transition may unfold again as Gen Z rises into leadership roles and begins managing an even younger generation of “AI-natives”. Anticipating and addressing these generational mismatches will be essential for long-term workforce sustainability. By incorporation of the insights from this study into workforce training and planning efforts, current and future stakeholders can disrupt this recurring cycle of generational misalignment and help rural communities build a more responsive and future-ready workforce.

### 4.5 Short-Term Fixes, Long-Term Neglect, and Willful Blindness

Limited-resource rural communities often struggle to afford the O&M of their wastewater treatment systems^90^ due to low population density and a reduced taxpayer base.^2^ In response, local private and public utilities may seek to cut their O&M costs simply to remain in business—often at the expense of quality, redundancy, and reliability. For example, an interviewee—with a regional development role—described a common band-aid solution used by skilled trade workers: *“They usually have two pumps inside of [septic tanks], and they alternate back and forth. If one goes down, they’ll just let it go and leave the one in there to work. Or if both of them break down, they’ll grab one out of a different [system], leave it with one [operating pump], so they can take [a pump] and go stick it in the [system] that didn’t have either one working.”* Such practices may be driven by inadequate training or limited awareness, but they also reflect deeper systemic issues, including lack of financial incentives, workforce fatigue, and structural inefficiencies. The same interviewee elaborated: *“I feel like sometimes things just are allowed to rock along, rock along, rock along until they finally hit a point where now you need a major fix. You need to [adapt] it to some other type of system even. And hopefully that system’s going to require less work from your operator. I’m all for efficiency, but sometimes I question whether it’s technical efficiency that’s foremost in their mind or if the operator just doesn’t really want the responsibility for doing anything. With a lot of this equipment, I feel like there’s still oversight that’s needed. They need to go out every day and make sure it’s working.”* Regardless of the underlying cause—whether stemming from lack of awareness, profit-driven decisions, or negligence—more effective regulation and consistent oversight could mitigate these issues, identify their root cause, and ensure accountability to prevent system failures. Accordingly, future policies need to shift from a *reactive* mindset to a *preventive*, systems-oriented approaches that can reduce operational costs and promote more reliable workforce practices (Table S5).

One such root cause—negligence—deserves closer attention, given its subtle yet far-reaching impact on infrastructure and WFD. Negligence is defined as the failure to exercise the level of care, skill, and knowledge expected of a competent agent, resulting in harm or risk, even when the agent believes they are acting correctly. This tendency often arises from a lack of awareness, outdated knowledge, or unrecognized risks, leading to unintended consequences despite good intentions.^91^ Neglect in the wastewater sector often manifests not through intentional harm but through uninformed decision-making, regulatory misalignment, and a failure to recognize the importance of high-level decision makers’ technical expertise in decentralized wastewater management. In Alabama’s Black Belt, multiple cases of neglect were reported by the interviewees. For instance, neglect through delegation illustrates how decision-makers distance themselves from infrastructure responsibilities by redirecting training efforts to technical staff. As one interviewee described: *“Trying to coordinate and educate is tough. They [elected officials] are going to see water and sewer as the topic of the whatever [training initiative], and they’re going to immediately say “Oh, that’s for Joe, he’s our water guy. He’ll deal with that”. In other words, they’re looking to pass on to whoever it might be that’s appropriate, not thinking that they themselves need to be involved with it at all, because they got a guy for that.”* While delegation is sometimes practical, in this case, it results in decision-makers disengaging from the very systems they are responsible for shaping through policies and budgets. Since it is not “Joe the water guy” but elected officials who vote on regulations, this knowledge gap contributes to policies that can be misaligned with technical and operational realities. One operator explained this disconnect starkly: *“It’s not just operators, it’s regulators. I hate to point fingers, but they [regulators] are not part of the solution right now. Even the regulations are being driven by somebody who doesn’t understand the technology. It’s disappointing sometimes when you see regulations that nobody realized were written more as a roadblock than a help because they didn’t have the technical expertise.”* This regulatory shortcoming ripples across other career pathways. Operators are forced to comply with impractical regulations that fail to reflect real-world conditions, wasting time on bureaucracy instead of focusing on system efficiency. Professionals must navigate conflicting design constraints that inhibit innovation. Academics exhaust valuable resources attempting to train decision-makers who ultimately fail to participate in the learning process.^92^ This misalignment between regulators and the remaining career pathways generates a feedback loop of ineffective oversight, professional frustration, and institutional inertia. Addressing this disconnect requires more than better delegation—it calls for active regulatory participation in technical training and cross-sector collaboration. Decision-makers must engage directly with the realities of DWSs, codeveloping practical, enforceable policies with professionals, operators, and academics. Only then can governance be aligned with system needs, preventing regulatory roadblocks, and strengthening the foundation for sustainable infrastructure and WFD.

However, beyond passive neglect and misalignment, a more troubling pattern often emerges—one rooted in willful blindness. In the context of wastewater infrastructure, willful blindness refers to the deliberate avoidance of acknowledging known risks and potential failures, even when there is sufficient information and capacity to address them.^93^ This willful inaction can have significant repercussions on all four career pathways and squander the already limited resources available to rural communities. One of the most striking examples of perceived regulatory failure comes from a community member who directly witnessed the consequences of limited oversight and the dismissal of technical expertise. His account reflects the deep mistrust that may emerge when agencies do not clearly communicate the basis of their decisions—leaving community members to believe that flawed projects were approved despite known risks. This mistrust, compounded by the lack of transparency, evolved into a deep resentment toward authorities. The following statement reflects this sentiment: *“You have [regulatory entity] in charge of this project, they allowed it knowing it would not work. What state agency, that is in the business of knowing the soil and the environment, will allow you to have a lagoon system with a spray field in this soil [impervious clayey soil]? [···] They allow $4.8 million to go down the drain knowing that they wouldn’t meet the percolation requirements. So, if I knew it wouldn’t work, you can’t tell me that they didn’t know. But they allowed it. I’ll never forget what the engineers said. He said [the regulatory entity] told him to go on and do the job, it’ll be alright. Knowing that this test [soil percolation test] needs to be provided for the agency before you even start on the project. That test was never done until the end of the project. [···] That’s another reason there’s very little trust in the agencies, because they have failed us at protecting the environment and the people.”* While regulatory agencies were the primary decision-makers in this failed $4.8 million project, responsibility also lies with the professional pathway. The engineers who executed the plan did so despite the absence of a required soil percolation test, raising serious questions about professional accountability and reinforcing the cycle of willful blindness. Whether due to pressure from regulatory agencies, financial incentives, or a lack of professional accountability, this inaction contributed to a system that was designed to fail. This instance of neglect raises a larger concern—when technical experts comply with flawed directives instead of challenging regulatory missteps, they become complicit in perpetuating the very workforce shortcomings and infrastructure failures that undermine public trust. This case underscores a broader institutional flaw: when regulators and professionals are allowed to evaluate their own decisions, conflicts of interest arise, reinforcing closed feedback loops and obstructing transparency. To disrupt this cycle, project oversight must be conducted by independent reviewers—not the same agencies or firms responsible for project approval or implementation. Without external checks, willful blindness becomes embedded in the system, allowing failures to repeat under the guise of technical or regulatory authority.

The findings of this study underscore the importance of addressing both short-term fixes and long-term systemic neglect in wastewater infrastructure management in limited-resource rural communities. Patterns of negligence, willful blindness, and inadequate regulation highlight the need for a shift toward more accountable and proactive oversight. A promising contrast to this pattern is the Pacific Region Infrastructure Facility (PRIF) strategy,^94^ which aims to break the “build-neglect-rebuild” cycle. Rather than allowing infrastructure systems to deteriorate due to unclear responsibilities and underfunded maintenance, the PRIF framework emphasizes routine upkeep through legal mandates, performance targets, and structured asset management plans. It prioritizes preventive maintenance and clarifies institutional roles to reduce the operational and financial burdens of recurring system failures. In contrast, Alabama’s Administrative Code for Decentralized Wastewater Management^22^ remains largely reactive once a system has been permitted and installed. While the code requires system approval and compliance upfront, ongoing oversight is minimal. For instance, Rule 420-3-1-.03 introduces penalties only after violations occur, and Rule 420-3-1-.32 mandates repairs only after a system failure is reported. Rule 420-3-1-.04 provides a general definition of maintenance but offers no enforceable schedules or inspection protocols. Although Rule 420-3-1-.16 tasks wastewater management entities with oversight responsibilities, it provides little guidance about proactive inspections or monitoring systems. This gap in regulatory design leaves communities vulnerable to costly system breakdowns that could have been prevented with a structured, anticipatory policy. Integrating insights from frameworks—such as PRIF^94^—with the cross-pathway analyses presented in this study could inform more robust and proactive O&M policies for DWSs in the U.S., especially in limited-resource rural contexts like Alabama’s Black Belt.

### 4.6 Access vs Enforcement: Imbalance Between Wastewater Compliance Policies and Community Realities

The American Bar Association^95^ highlights how fines and legal financial obligations disproportionately burden low-income communities, trapping individuals in cycles of debt rather than promoting compliance. This is particularly relevant in Alabama’s Black Belt, where rigid enforcement strategies fail to address the root causes of wastewater violations—namely, the lack of financial resources and infrastructure. Rather than improving compliance, punitive fines risk worsening the crisis by penalizing residents for circumstances beyond their control. Despite these challenges, regulators hold divergent views on how to enforce compliance. Some regulators insist on strict regulatory enforcement, applying uniform penalties under the assumption that consistency drives accountability. One regulator explained this approach: *“We inspect them regularly, and they have to report different parameters regularly so that we can monitor whether they’re meeting their water quality requirements. But as far as Black Belt, they’re like anybody else. If they’re not meeting their water quality standards, then we undertake enforcement action.”* However, not all regulators share this one-size-fits-all mindset. Those deeply embedded in the community often recognize that punitive measures alone do not address the underlying financial and infrastructural barriers to compliance. For example, one regulator with such ties with the community described how economic hardship—not homeowner’s negligence—is often the cause of sanitation violations: “*The reason you are getting straight pipes for the person who flush their toilet, and the sewage come out on the surface, is because they can’t afford the system. If they can’t afford a system and sewage is on the ground and we go out and we write a citation, they still can’t do anything with it. They have no money. [···] So, we don’t write citations, not right now.”* This divergence reflects a larger issue within the decentralized wastewater workforce: the disconnect between standardized regulatory practices and the lived realities of limited-resource communities. While regulators are often perceived as external enforcers, many are themselves members of the very communities they serve. Their proximity fosters a nuanced understanding of local conditions and enables more context-sensitive approaches to enforcement. Rather than viewing this closeness as a liability, it should be seen as an asset—one that offers an opportunity to build more responsive regulatory practices. Enforcement remains a critical function of the regulatory career pathway and a cornerstone of infrastructure development,^96^ but the means of enforcement matter. Regulators with deep community ties often favor guidance, flexibility, and tailored support—approaches better aligned with the financial and infrastructural constraints of rural areas. Bridging this divide between detached uniformity and embedded flexibility is essential for advancing effective and sustainable sanitation solutions in contexts like Alabama’s Black Belt.

This community-based perspective is not limited to regulators; operators from the skilled trade career pathway who work directly with noncompliant systems also advocate for a more solution-oriented regulatory approach. Like community-embedded regulators, these frontline workers recognize that fines alone do not resolve structural wastewater issues. Instead, they advocate for a more proactive, solution-oriented approach—one that emphasizes support and problem-solving over punishment. As one operator explained: *“I think the government has plenty of people, expertise and money. So, if you’re going to impose a new regulation [about enforcement], then solve it before you pull the trigger on the regulation. They’re smart enough to do it, but they need to provide more solutions than just regulations.”* Operators often find themselves between two worlds: the demands of regulators and the financial limitations of the communities they serve. They witness firsthand how rigid enforcement, in the absence of viable alternatives, leads to recurring violations and persistent system failures. When enforcement actions are not accompanied by technical or financial assistance, noncompliant households remain trapped in cycles of citations and collapse. This frustration among operators underscores the need for a shift from hostile enforcement to a more facilitative, solution-driven regulatory framework and highlights the rich insights that skilled trade workers can provide to improve current policies.

Academics also contribute to the debate between community-embedded and strictly enforcement-oriented regulators by offering a broader system-level perspective on compliance. While regulators and operators focus on immediate enforcement challenges—whether to penalize or not—academics reframe the issue as a structural failure that requires long-term, systemic change. They argue that the debate should not be reduced to enforcement versus nonenforcement but rather reframed as a question of how to ensure compliance in ways that maintain public health standards without overburdening limited-resource rural communities. A university professor with research experience in rural infrastructure explains: *“Regulations are the minimum standards. We can always design something that works better than what’s required by regulations. We never do. From that perspective, regulations are the minimum standard [that] we, as a society, are not willing to give up just because somebody can’t afford it. We have notions of environmental health and public health, and that minimum standard is going to ensure the continuation of environmental and public health. Right now, those people aren’t meeting minimum standards. They’ve got sewage flowing down the roadways. So almost anything can be an improvement, but we’ve got to bring it up to [the standards], not to [what people can afford]. [···]. But, when we have situations [where people can’t afford it] then agencies have to bridge the gap between where we are now and where we need to be through funding, not the community. We have demonstrated that we can allocate money, so let’s use that money to bridge the gap to bring it up to standard.”* This academic perspective expands on the concerns raised by community-embedded regulators and skilled trade workers but shifts the conversation toward structural reform. Rather than merely modifying enforcement strategies at the margins, academics call for the underlying systems to be restructured so that compliance is not just legally required but also practically achievable. Regulations must reflect a commitment to public and environmental health, but achieving those standards should not fall solely on communities that lack the resources to comply. Instead, federal and state agencies must take responsibility for bridging the financial and infrastructural gaps using available funding to support sustainable compliance.

This debate is not unique to Alabama’s Black Belt but reflects a broader challenge faced by limited-resource communities across the country. Regulatory frameworks often impose compliance standards for the whole state without accounting for the socio-technical barriers that prevent their achievement in some communities.^97^ Findings from this study suggest that a regulatory approach that is neither strictly punitive nor overly permissive is needed in these contexts. Instead, enforcement strategies should be integrated with the economic, technical, and policy realities specific to each limited-resource community to enable long-term and appropriate—rather than uniform—access to sanitation solutions. For example, aligning infrastructure priorities with local needs through socio-technical integration^68^ would allow for the creation of more context-specific policies. In this context, giving municipalities a seat at the table when regulatory standards are set may help. As the actors closest to on-the-ground realities, municipalities can ensure that compliance expectations take into consideration community realities, avoiding a top-down, one-size-fits-all approach.^98^ By acknowledging the intersection of the four workforce career pathways (see Table S5 in the Supporting Information), as well as infrastructure and financial constraints, this research provides insights that can inform more adaptive, community-centered approaches to wastewater governance—both in Alabama’s Black Belt and in similarly limited-resource rural regions.

## 5 Outlook and Future Research

The persistent failures of DWSs documented in this study are not the result of a lack of effort but rather a consequence of misaligned and fragmented workforce pathways. Especially in limited-resource rural communities, the isolation of critical actors in academic, regulatory, professional, and skilled trade pathways continues to undermine the sustainability and effectiveness of DWSs. In response, this study proposes a novel approach to WFD that goes beyond providing new tools—offering instead a conceptual shift to identify the root causes of challenges that emerge at the intersection of these four pathways. By clarifying how stakeholders’ responsibilities are shared, where breakdowns occur, and where targeted interventions are most urgently needed, this work answers both decade-long^21^ and recent calls^5,19,20^ for comprehensive WFD strategies. To the best of the authors’ knowledge, this is the very first scholarly article to holistically examine both workforce shortages and shortcomings in the context of DWSs, advancing a deeper understanding of the interconnected nature of the wastewater workforce. Moreover, to strengthen the analytical foundation of this work, we draw policy insights from sectors where workforce development has been more extensively studied, including education, transportation, and manufacturing. Integrating these cross-sectoral perspectives allowed us to contextualize the challenges facing the DWS workforce and reveal valuable parallels in how other sectors approach recruitment, training, and retention. This study culminates in targeted policy recommendations, including proposed amendments to specific articles in the Alabama Administrative code, directly contributing to the ongoing efforts to strengthen DWS implementation in limited-resource rural communities and to advance progress toward national SDG 6. Although the findings of this study are grounded in Alabama’s Black Belt, the proposed cross-pathway approach is designed to be both flexible and transferable to other limited-resource rural communities in the U.S.—such as rural Alaska, Appalachia, Navajo Nation, and Texas Colonias. These communities, although differing in culture and local histories, often face overlapping structural constraints, including extreme geological conditions, high poverty rates, and limited government capacity. We fully recognize that each community brings unique socio-technical challenges, institutional norms, and workforce dynamics that must be carefully considered. However, the use of semistructured interviews in the design of this study—coupled with theoretical thematic analysis guided by a pre-established framework^18^—enables this approach to remain responsive to local conditions while capturing insights from stakeholders across the four career pathways.

We encourage future research to apply this cross-pathway approach in other limited-resource rural communities to generate context-specific WFD strategies and examine whether similar patterns of cross-pathway challenges emerge. In addition to its geographic adaptability, the cross-pathway approach is applicable in other decentralized infrastructure systems^2^—such as rural broadband, off-grid energy, and community-based healthcare—that similarly suffer from siloed WFD efforts and fragmented institutional support. When applying this cross-pathway approach in future studies across different communities and infrastructure sectors, researchers are encouraged to (1) expand the cross-sectoral perspective to further bridge wastewater studies with insights from adjacent fields to strengthen policy recommendations and strategies for wastewater workforce development and (2) broaden the spectrum of stakeholders and tailor the participant selection to reflect context-specific dynamics. This selection includes incorporating pivotal roles, such as tribal governments, community organizers, and private-sector developers, especially in regions where these actors play critical roles in infrastructure planning or WFD. Additionally, future work should strive for a more balanced distribution of interviewees across the four career pathways and examine whether such balance reinforces, challenges, or nuances the insights generated from this study.

Quantitative verifications of this cross-pathway approach would also contribute to its robustness. Future research should aim to quantitatively validate the distinctiveness of cross-pathway versus pathway-specific challenges by developing quantitative survey instruments grounded in this study’s qualitative findings. Analytical techniques, such as measurement invariance testing,^99^ can determine whether challenges are conceptualized similarly across career pathways, while multigroup analysis^100^ can reveal whether their impacts differ significantly between groups. Such analyses would empirically strengthen the argument that decentralized workforce challenges span multiple career pathways rather than being isolated within a single pathway. In parallel, future research should build on this study’s direction by investigating how cross-pathway challenges concretely impact workforce attraction and retention. Similar to the authors’ current work, applying predictive empirical methods—such as Partial Least Square Structural Equation Modeling^101^—would allow researchers to link qualitative insights to more measurable workforce outcomes, offering more actionable guidance for workforce planning and policy design.

The current US-EPA’s Decentralized Wastewater Workforce Framework^5^ could also be improved to reflect the realities of decentralized wastewater management more accurately. First, future research should consider disaggregating the regulatory pathway, which currently combines distinct roles, such as policymakers, grant writers, and permitting officials, under a single category. Disaggregating these roles would enable a more granular understanding of how different regulatory functions shape workforce challenges and their intersections with other pathways. Second, there is a need to formally recognize community members as core actors in the workforce structure rather than treating them solely as external stakeholders. In DWSs, especially in rural settings, community members frequently engage in informal system monitoring, minor maintenance, advocacy, and decision-making—often because DWSs are located on or adjacent to their property. Including community members as a distinct stakeholder group would better align WFD strategies with the realities on the ground, particularly in limited-resource rural settings where residents function as de facto infrastructure managers compared to their more distanced urban counterparts.

Additionally, the current US-EPA’s Decentralized Wastewater Workforce Framework^5^ implicitly assumes a horizontal structure among the four career pathways, treating them as if they operate on an equal footing. However, this structure overlooks the reality that significant power dynamics exist between stakeholders, which can heavily influence the implementation of workforce solutions. Different stakeholders bring distinct priorities, interests, and levels of influence, often creating tensions or misalignments that shape decision-making processes. To address this gap, ongoing work by the authors applies the Matrix of Alliances and Conflicts: Tactics, Objectives, and Recommendations (MACTOR) analysis to systematically map stakeholder alignments, conflicts, and power relations. This effort aims to strengthen future WFD strategies by making stakeholder relationships and their practical impacts more transparent.

Finally, while this study identified critical gaps in existing codes and policies and proposed potential solutions specific to Alabama, future research should advance this work by codeveloping training materials and policy recommendations in direct partnership with stakeholders from each pathway. Advancing cross-pathway solutions is essential for meeting SDG 6, but the stakes extend far beyond metrics and targets. At its core, this work is about removing raw sewage from people’s lawns, restoring dignity to limited-resource rural communities, and ensuring that the infrastructure systems meant to provide basic services and protect public health do not fail the very people who need them most.

## Supplementary Material



## Notes

Characteristics of the four career pathways in decentralized wastewater infrastructure; study rigor metrics and COREQ matrix; interviewee demographics and representativeness; identified pathway-specific challenges; summary of the cross-pathway challenges identified and the implication for each pathway (PDF)
