# Supplementary material for: Shared Failures: Uniting Four Career Pathways to Overcome Decentralized Wastewater Workforce Challenges in Limited-Resource Rural Communities
Source: ACS ES T Water. 2025 Nov 14;5(12):7475–91. doi: 10.1021/acsestwater.5c00978 (PMC12706779; doi:10.1021/acsestwater.5c00978)
Supplement: Supplementary file 1 [file ew5c00978_si_001.pdf]

Shared Failures: Uniting Four Career Pathways to Overcome Decentralized Wastewater Workforce Challenges in Limited-Resource Rural Communities

Table of Contents

1. Characteristics of the Four Career Pathways in Decentralized Wastewater Infrastructure..... 2
2. Study Rigor Metrics and COREQ Matrix 4
3. Interviewee Demographics and Representativeness.. 8
4. Identified Pathway-Specific Challenges... 12
5. Summary of the Cross-Pathway Challenges Identified and the Implication for Each Pathway 15
6. References 20

Number of Tables: 5

1. Characteristics of the Four Career Pathways in Decentralized Wastewater Infrastructure

Table S1. Characteristics of the four career pathways, based on US-EPA (2021)

Characteristic	***Academic***	***Regulatory***	***Professional***	***Skilled Trade***
Sector(s)	Public/Private (Universities, Training Centers)	Public/private (Government Agencies, private utilities)	Private (Engineering, Consulting, Sales)	Private (Manufacturing, Installation, Maintenance)
Responsibilities	Conducts research, educates students, develops curriculum, fosters expertise in decentralized wastewater	Oversees compliance, inspects wastewater systems, enforces environmental and health regulations	Designs decentralized wastewater systems, conducts environmental analysis, provides consulting services	Installs, repairs, and maintains decentralized wastewater systems, operates heavy equipment for excavation
Example Roles	Engineering Professor, Environmental Science Professor, Microbiologist	Environmental Health Specialist, Compliance Manager, Occupational Health and Safety Technician	Civil Engineer, Hydrologist, Soil Scientist, Sales Manager	Septic System Installer, Electrician, Pipelayer, Equipment Operator
Education & Training Required	Master’s or Ph.D. in Environmental Science, Engineering, Microbiology	Bachelor's in Environmental Science, Public Health, or related field	Bachelor’s or Master’s in Engineering, Environmental Science, Hydrology, or Business (for Sales)	High school diploma + technical training/apprenticeship
Key Skills & Competencies	Research, Teaching, Technical Knowledge	Policy Knowledge, Inspection, Risk Assessment	Technical Analysis, GIS Mapping, Engineering Design, Soil & Water Testing	Hands-on Installation, Equipment Operation, Troubleshooting
Typical Employers	Universities, Research Institutions	EPA, State & Local Health Departments, Environmental Agencies	Consulting Firms, Environmental Agencies, Private Companies	Construction Firms, Septic System Service Providers, Manufacturing Companies, Public/Private Utilities

Characteristic	***Academic***	***Regulatory***	***Professional***	***Skilled Trade***
Certifications & Licenses	N/A	Registered Environmental Health Specialist (REHS), Occupational Safety Certification	Professional Engineer (PE), Certified Hydrologist	Septic System Installer License, Electrician License, Heavy Equipment Operator Certification
Growth Potential & Job Outlook	Low supply of faculty with decentralized expertise, demand for research to support workforce development	High demand due to environmental concerns and regulatory requirements	Moderate demand, driven by technology adoption and environmental policies	High demand, rapid growth in decentralized system installations, shortage of skilled workers
Average Salary Range	$70K - $100K	$50K - $75K	$70K - $120K	$40K - $70K

2. Study Rigor Metrics and COREQ Matrix

The researcher who conducted data collection has extensive experience in qualitative research, having led multiple studies in this domain. To minimize interviewer bias, the questions were designed to be neutral and carefully phrased so as not to imply the existence of a challenge. Interviews were conducted privately, with only the researcher conducting data collection and interviewees present.

Out of the 32 rigorous qualitative research reporting criteria in the Consolidated criteria for reporting qualitative research (COREQ) matrix,[2] 27 were adequately addressed in this study and 5 were deemed irrelevant (see Table S2). Furthermore, rigor in qualitative research is characterized by two main components: validity and reliability.[3] In addition to meeting intercoder reliability requirement, we addressed validity through three key components: authenticity, plausibility, and criticality. [3] The analysis drew from both the interview transcripts and memos taken by the researcher during the interviews. A preliminary rapport was established with some participants prior to data collection by engaging with them directly and sharing the study's objectives. Authenticity is maintained through the use of theoretical thematic analysis,[4] which is innately inductive and based on insights directly derived from the interviewees and their lived experiences. In terms of plausibility, the findings were critically analyzed, and the premise for the analysis is grounded in existing literature through triangulation across multiple sources, which strengthens the credibility of the results.[5,6]Additionally, the criticality of the study is evident in the way it challenges the reader to reconsider the impact of only addressing pathway-specific challenges. By exploring how these challenges intersect, the study compels a more holistic view of workforce development (WFD), providing evidence of the ripple effect caused by isolating one pathway over others.

Table S2. Completed COREQ Matrix[2]

No. Item	Guide questions/description	Reported in section
**Domain 1: Research team and reflexivity**		
Personal Characteristics		
**1. Interviewer/facilitator**	Which author/s conducted the interview or focus group?	Author contribution section
**2. Credentials**	What were the researcher's credentials? E.g. PhD, MD	Title Page
**3. Occupation**	What was their occupation at the time of the study?	Title Page
**4. Gender**	Was the researcher male or female?	Irrelevant for this study
**5. Experience and training**	What experience or training did the researcher have?	SI Section 2
Relationship with participants		
**6. Relationship established**	Was a relationship established prior to study commencement?	SI Section 2
**7. Participant knowledge of the interviewer**	What did the participants know about the researcher? e.g. personal goals, reasons for doing the research	SI Section 2
**8. Interviewer characteristics**	What characteristics were reported about the interviewer/facilitator? e.g. Bias, assumptions, reasons and interests in the research topic	SI Section 2
**Domain 2: study design**		
Theoretical framework		
**9. Methodological orientation and Theory**	What methodological orientation was stated to underpin the study? e.g. grounded theory, discourse analysis, ethnography, phenomenology, content analysis	Section 2.3
Participant selection		
**10. Sampling**	How were participants selected? e.g. purposive, convenience, consecutive, snowball	Section 2.2
**11. Method of approach**	How were participants approached? e.g. face-to-face, telephone, mail, email	Section 2.2

No. Item	Guide questions/description	Reported in section
**12. Sample size**	How many participants were in the study?	Section 2.2
**13. Non-participation**	How many people refused to participate or dropped out? Reasons?	NA
Setting		
**14. Setting of data collection**	Where was the data collected? e.g. home, clinic, workplace	Section 2.2
**15. Presence of non-participants**	Was anyone else present besides the participants and researchers?	SI Section 2
**16. Description of sample**	What are the important characteristics of the sample? e.g. demographic data, date	Section 2.2 and Table S3
Data collection		
**17. Interview guide**	Were questions, prompts, guides provided by the authors?	Available from the corresponding author upon reasonable request.
**18. Repeat interviews**	Were repeat interviews carried out? If yes, how many?	NA
**19. Audio/visual recording**	Did the research use audio or visual recording to collect the data?	Section 2.2
**20. Field notes**	Were field notes made during and/or after the interview or focus group?	Section 2.2
**21. Duration**	What was the duration of the interviews or focus group?	Section 2.2
**22. Data saturation**	Was data saturation discussed?	Section 2.2
**23. Transcripts returned**	Were transcripts returned to participants for comment and/or correction?	NA
**Domain 3: analysis and findings**		
Data analysis		
**24. Number of data coders**	How many data coders coded the data?	Section 2.3
**25. Description of the coding tree**	Did authors provide a description of the coding tree?	Section 2.3, 3.1 and 3.2
**26. Derivation of themes**	Were themes identified in advance or derived from the data?	Section 2.3
**27. Software**	What software, if applicable, was used to manage the data?	Section 2.3

No. Item	Guide questions/description	Reported in section
**28. Participant checking**	Did participants provide feedback on the findings?	NA
Reporting		
**29. Quotations presented**	Were participant quotations presented to illustrate the themes/findings? Was each quotation identified? e.g. participant number	Due to the small nature of the studied community, interviewee numbers were not included in the reporting to guarantee participant anonymity. However, the general role of the interviewee was given before each quote.
**30. Data and findings consistent**	Was there consistency between the data presented and the findings?	Section 3 and Section 4
**31. Clarity of major themes**	Were major themes clearly presented in the findings?	Section 3 and Section 4
**32. Clarity of minor themes**	Is there a description of diverse cases or discussion of minor themes?	Section 3 and Section 4

3. Interviewee Demographics and Representativeness

A brief, structured demographic questionnaire was provided after each interview to minimize any potential discomfort associated with responding to demographic questions in real-time. Interviewees were also given the option to decline any demographic question they preferred not to answer.

Table S3. Interviewee Demographics

Interviewee #	Job Title	Career Pathway	Gender	Race/Ethnicity	Age Range	Native Language	Income Range	Years Of Experience
**1**	Emeritus professor	Academic	Male	White	65 and over	English	Over 95,000	More than 30 years
**2**	Associate Professor	Academic	Male	White	55-to-64	English	Over 95,000	More than 30 years
**3**	Riverkeeper	Community	Male	White	35-to-44	English	51,000-to-70,000	11-to-20 years
**4**	President	Community	Male	Black or African American	55-to-64	English	20,000-to-30,000	6-to-10 years
**5**	Executive Director	Community	Female	Black or African American	35-to-44	NA	36,000-to-50,000	11-to-20 years
**6**	Senior Planner	Community	Male	Black or African American	45-to-54	English	71,000-to-95,000	21-to-30 years
**7**	Environmental Engineer	Professional	Male	White	65 and over	English	51,000-to-70,000	More than 30 years
**8**	Vice President of Business Development	Professional	Male	White	55-to-64	English	Over 95,000	More than 30 years

Interviewee #	Job Title	Career Pathway	Gender	Race/Ethnicity	Age Range	Native Language	Income Range	Years Of Experience
**9**	Business development	Professional	Male	White	65 and over	English	Over 95,000	More than 30 years
**10**	CEO Emeritus	Professional	Male	White	65 and over	English	Over 95,000	More than 30 years
**11**	Staff Scientist	Professional	Male	White	35-to-44	English	51,000-to-70,000	11-to-20 years
**12**	Staff Attorney	Professional	Female	White	55-to-64	English	Over 95,000	21-to-30 years
**13**	Founding Principal	Professional	Male	White	65 and over	English	Over 95,000	More than 30 years
**14**	Wastewater Specialist	Professional	Male	White	55-to-64	English	51,000-to-70,000	More than 30 years
**15**	President	Professional	Male	White	65 and over	English	Over 95,000	More than 30 years
**16**	National Sales Manager - Municipal & Commercial Systems	Professional	Male	White	55-to-64	English	Over 95,000	More than 30 years
**17**	Operations Manager	Professional	Male	White	55-to-64	NA	Over 95,000	More than 30 years
**18**	Engineer	Professional	Male	White	45-to-54	English	Over 95,000	21-to-30 years
**19**	Sales Engineer	Professional	Male	White	35-to-44	English	Over 95,000	6-to-10 years
**20**	Director	Regulatory	Female	Black or African American	65 and over	English	Over 95,000	More than 30 years

Interviewee #	Job Title	Career Pathway	Gender	Race/Ethnicity	Age Range	Native Language	Income Range	Years Of Experience
**21**	Grant Administrator	Regulatory	Female	White	45-to-54	English	Over 95,000	21-to-30 years
**22**	Senior Advisor	Regulatory	Male	White	65 and over	English	Over 95,000	More than 30 years
**23**	Alabama State Coordinator	Regulatory	Female	White	55-to-64	English	71,000-to-95,000	21-to-30 years
**24**	Director	Regulatory	Male	Latin/Hispanic	45-to-54	English	Over 95,000	21-to-30 years
**25**	President	Regulatory	Female	Black or African American	65 and over	English	20,000-to-30,000	21-to-30 years
**26**	Director	Regulatory	Male	White	65 and over	English	Over 95,000	More than 30 years
**27**	Executive Director	Regulatory	Male	White	35-to-44	English	Over 95,000	21-to-30 years
**28**	Executive Director	Regulatory	Female	Black or African American	55-to-64	NA	Over 95,000	More than 30 years
**29**	Program Analyst	Regulatory	Female	Black or African American	25-to-34	English	Over 95,000	11-to-20 years
**30**	Retired	Regulatory	Male	White	65 and over	English	71,000-to-95,000	More than 30 years
**31**	Director of Community and Economic Development	Regulatory	Female	White	55-to-64	English	71,000-to-95,000	21-to-30 years
**32**	Local government	Regulatory	Male	Black or African American	65 and over	English	Over 95,000	6-to-10 years

Interviewee #	Job Title	Career Pathway	Gender	Race/Ethnicity	Age Range	Native Language	Income Range	Years Of Experience
**33**	General Manager	Skilled Trade	Male	White	45-to-54	English	Over 95,000	21-to-30 years
**34**	Operator	Skilled Trade	Male	White	25-to-34	English	51,000-to-70,000	1-to-5 years
**35**	Wastewater Superintendent	Skilled Trade	Male	White	45-to-54	English	71,000-to-95,000	11-to-20 years

4. Identified Pathway-Specific Challenges

Table S4. Sample of the Coding Dictionary for the Pathway-Specific Challenges

Code	Description	*Example*
**ACADEMIC CAREER PATHWAY**		
Certification Requirements Misaligned with Real Job Responsibilities	Candidates are required to pass certifications testing knowledge unrelated to the actual tasks they will perform on the job.	*In Alabama, the problem they're having now is they've made it so hard to become one [operator]. It's gotten to the point to where they require you to know so much knowledge about stuff that has nothing to do with what you do.*
Exclusion of the Community from Wastewater Sector Research and Development Efforts	Community members are not adequately involved or consulted in wastewater infrastructure decisions due to both ineffective engagement initiatives and a lack of community receptiveness.	*No one came in and actually had a discussion with the community to teach them, or train them, or inform them on how they need to handle things. In the town meetings, it's more or less a dictatorship telling [the residents], 'This is what we [the utility or regulatory entity] are going to do.'*
**PROFESSIONAL CAREER PATHWAY**		
Lack of Capacity and Competitiveness Incentives Resulting in Reduced Entrepreneurial Initiatives	The unprofitable perception of the wastewater field and the economic hardship of local communities portray the wastewater market as a poor choice for an entrepreneurial venture.	*Pretty much everybody feels like sewer is not profitable at all. Sewer is where you just run into to just all sorts of financial mess because it does not make money. It just doesn't. And why would you want to get involved with something that really doesn't make money.*

Code	Description	*Example*
In-House Training Becoming a Necessity Due to Irrelevant Training	Employers are forced to provide their own training to new hires costing companies time and money.	*When we're hiring engineers, we're measuring what their aptitude is. Then, we're spending a year training them ourselves because they don't come out of school with any skills relevant to what we and what the industry really needs. We have invested a lot of time and money in some of them, and they've gone off to much bigger jobs beyond [company name] because suddenly, they've got this training*
**<u>REGULATORY CAREER PATHWAY</u>**		
Disconnect between external regulation and local realities	Regulatory enforcement actions that lack community input and fail to reflect local conditions, often emphasizing punishment over practical solutions.	*Now, we inspect them on a regular basis, and they have to report different parameters on a regular basis so that we can monitor whether they're meeting their water quality requirements. But as far as Black Belt, they're like anybody else. If they're not meeting their water quality standards, then we undertake enforcement action.*
Rigid Laws Restrict Innovation and Enforce Prescriptive Attitude	Strict regulations prioritize rigid, one-size-fits-all solutions over adaptive and innovative approaches, limiting flexibility in wastewater management.	*The biggest problem with the regulatory business* […] *is that regulators like to have a book with all the pages. It says on this page that if you got this condition, [then] this is what you have to do. That's called prescriptive regulation. Every problem has a prescription to fix it.* […] *So many of us have been fighting for the last two or three decades to get regulations changed from prescriptive to performance based.*
**<u>SKILLED TRADE CAREER PATHWAY</u>**		
Difficulty Retaining Workers	Generational gaps, and limited pay/benefits make it difficult to retain	*Because once you get them certified, then all of a sudden, the next community over who has more dollars*

Code	Description	*Example*
	a stable wastewater workforce, especially in small systems.	*might snatch them away from you. You spent all the money training them, now they're a higher qualified person and they can go to the next major town to get more money.*
Difficulty of skilled trade workers to pass certification exams	Challenges faced by skilled trade workers in successfully passing required certification exams due to gaps in prior education	*A lot of times you get kids that [taking tests is] just not their thing. You put a motor in front of them, they can take it apart and put it back together again. But you want them to read, write, and do math, [they will struggle]. So, a lot of the tutoring [needed] is in math.*

5. Summary of the Cross-Pathway Challenges Identified and the Implication for Each Pathway

Table S5. Cross-Pathway Challenges in Alabama's Black Belt

Emergent Cross-pathway Challenge	Involved Pathway	Interactions with Other Pathways	Proposed Targeted Recommendation
**Lack of Structural Support for O&M Entrepreneurial Endeavors**	Professional	Professionals' perception of low profitability is reinforced by regulatory rigidity, discouraging entrepreneurial initiatives and leaving skilled trades without clear growth paths.	Professionals must reframe the narrative around wastewater profitability and advocate for innovative business models such as rural entrepreneurship.
	Regulatory	Regulators impose prescriptive standards that stifle innovative business models, indirectly confirming professionals' negative views and limiting opportunities for skilled trades. Disconnection between regulatory intent and professionals' perceptions deepens the entrepreneurial risk landscape.	Shift from prescriptive to performance-based standards to foster entrepreneurial ventures.
	Skilled Trade	Skilled trade workers, while sharing the sentiment of unprofitability, are passive recipients of a job market lacking entrepreneurial support.	Push for initiatives that integrate skilled trades into sustainable, community-based business models.
**Siloed Academic, Regulatory, and Professional Pathways Hindering Skilled Trade Development**	Academic	Academics develop curricula based on centralized models, leading to a disconnect with industry needs and forcing professionals to invest in additional training.	Revise curricula to incorporate decentralized wastewater management and align training with industry needs.
	Regulatory	Regulators have the potential to mediate between theoretical academic training and	Act as mediators to ensure academic programs reflect requirements outlined by

Emergent Cross-pathway Challenge	Involved Pathway	Interactions with Other Pathways	Proposed Targeted Recommendation
		practical industry demands, yet current silos hinder effective collaboration.	professionals while safeguarding skilled trade workers' rights on the job.
	Professional	Professionals face a mismatch between academic preparation and real-world demands, resulting in costly in-house training and inefficiencies.	Advocate for educational reforms that reduce the need for extensive in-house retraining.
	Skilled Trade	Skilled trade workers rely on academic instruction but experience significant gaps between theoretical knowledge and practical skills needed on the job.	Collaborate with academia and industry to develop practical, job-relevant skill sets.
**Cultural Barriers to Development Efforts: Breaking Through Professional, Regulatory, and Social Resistance**	Professional	Professionals often underestimate local knowledge, leading to conflicts and mistrust when implementing infrastructure projects in culturally sensitive contexts.	Enhance community engagement and develop cultural competence to build local trust.
	Regulatory	Regulators' top-down, prescriptive approaches exacerbate community resistance and hinder collaborative problem-solving with local stakeholders.	Adopt participatory approaches that incorporate local voices in decision-making.
	Academic	Academics are increasingly aware of the need for community engagement, and by actively co-developing solutions with local communities, they can shift from extractive practices to genuine collaborative partnerships.	Foster community-engaged research and co-create solutions with local stakeholders.

**Emergent Cross-pathway Challenge**	**Involved Pathway**	**Interactions with Other Pathways**	**Proposed Targeted Recommendation**
	Skilled Trade	Skilled trades are indirectly impacted by cultural miscommunications and the resulting disconnect between decision-makers and local communities.	Work with professionals to ensure respectful, community-informed implementation of infrastructure efforts.
**Generational Divide in Gen Z's Workforce Expectations and Realities**	Professional	Older professionals' rigid practices and reluctance to adapt create barriers for integrating Gen Z, affecting workforce continuity and innovation.	Modernize workplace practices and mentoring to better integrate Gen Z talent.
	Regulatory	Regulatory bodies maintain outdated work policies and communication methods that fail to attract or retain young talent in the workforce.	Update policies and outreach strategies to resonate with younger workforce expectations.
	Academic	Academics champion co-creative design methods, enabling professionals to work with Gen Z applicants in co-developing inclusive workplaces that accommodate both senior employees and newer workforce members.	Introduce collaborative methodologies and training programs that foster generational inclusivity, guiding professionals in bridging gaps between senior staff and Gen Z entrants.
	Skilled Trade	Skilled trade workers are caught in the generational divide, experiencing the impact of outdated practices while lacking a role in shaping change.	Work with industry and regulators to create accessible pathways for younger workers.
**Short-Term Fixes, Long-Term Neglect, and Willful Blindness**	Academic	Academia's struggle to effectively educate decision-makers contributes to a disconnect in technical oversight, reinforcing short-term fixes over sustainable solutions.	Improve engagement with decision-makers to ensure technical training is effective and relevant.

**Emergent Cross-pathway Challenge**	**Involved Pathway**	**Interactions with Other Pathways**	**Proposed Targeted Recommendation**
	Regulatory	Regulators enforce policies based on limited technical expertise, resulting in reactive measures that fail to address long-term infrastructure needs.	Invest in capacity-building and proactive external oversight to move from reactive fixes to preventive strategies.
	Professional	Professionals, by complying with flawed directives, perpetuate a cycle of reactive maintenance and systemic neglect.	Strengthen accountability and promote preventive design and maintenance practices to counter short-termism.
	Skilled Trade	Skilled trades are left to manage the fallout of short-term fixes, lacking the authority or resources to drive systemic change.	Advocate for policies that prioritize long-term system reliability over temporary solutions.
**Access vs. Enforcement: Imbalance Between Wastewater Compliance Policies and Community Realities in Limited-Resource Rural Communities**	Academic	Academics advocate for structural reforms that challenge existing punitive measures, aiming to reframe compliance through systemic change.	Propose systemic reforms and integrated funding models that support sustainable compliance.
	Regulatory	Regulatory bodies are split between strict equality-based enforcement and more flexible equity-based approaches, and this divide also draws in the other pathways, which must navigate or respond to these conflicting regulatory visions.	Transition to flexible enforcement that joins accountability with community support by guiding residents towards the relevant entities for improvement.
	Professional	Professionals face challenges reconciling rigid enforcement with local economic realities, which undermines operational effectiveness.	Push for enforcement coupled with financial and technical support to reflect local realities.

Emergent Cross-pathway Challenge	Involved Pathway	Interactions with Other Pathways	Proposed Targeted Recommendation
	Skilled Trade	Skilled trade workers bear the consequences of enforcement without adequate support, remaining largely passive in policy debates.	Demand enforcement models that provide alternatives to punitive measures and support operational efficiency.

6. References

(1) US-EPA. *Pipeline to a Sustainable Workforce: A Report on Decentralized/Onsite Wastewater Occupations*; 2021. https://www.epa.gov/septic/pipeline-strong-workforce-decentralizedonsite-wastewater-occupations (accessed 2025-06-24).

(2) Tong, A.; Sainsbury, P.; Craig, J. Consolidated Criteria for Reporting Qualitative Research (COREQ): A 32-Item Checklist for Interviews and Focus Groups. *International Journal for Quality in Health Care* **2007**, *19* (6), 349–357. https://doi.org/10.1093/INTQHC/MZM042.

(3) Phelps, A. F.; Horman, M. J. Ethnographic Theory-Building Research in Construction. *J Constr Eng Manag* **2010**, *136* (1), 58–65. https://doi.org/10.1061/(ASCE)CO.1943-7862.0000104.

(4) Braun, V.; Clarke, V. Using Thematic Analysis in Psychology. *Qual Res Psychol* **2006**, *3* (2), 77–101. https://doi.org/10.1191/1478088706qp063oa.

(5) Taylor, J. E.; Asce, M.; Carrie, ; Dossick, S.; Garvin, M. Meeting the Burden of Proof with Case-Study Research. *J Constr Eng Manag* **2010**, *137* (4), 303–311. https://doi.org/10.1061/(ASCE)CO.1943-7862.0000283.

(6) Gomes Araújo, L.; Lucko, G. Best Practices for Case Studies in Construction Engineering and Management Research. *J Constr Eng Manag* **2022**, *148* (8), 04022062. https://doi.org/10.1061/(ASCE)CO.1943-7862.0002312.
